# Barrier Membranes for Guided Bone Regeneration (GBR): A Focus on Recent Advances in Collagen Membranes

**DOI:** 10.3390/ijms232314987

**Published:** 2022-11-29

**Authors:** Yanru Ren, Lu Fan, Said Alkildani, Luo Liu, Steffen Emmert, Stevo Najman, Denis Rimashevskiy, Reinhard Schnettler, Ole Jung, Xin Xiong, Mike Barbeck

**Affiliations:** 1 Clinic and Policlinic for Dermatology and Venereology, University Medical Center Rostock, 18057 Rostock, Germany; 2 BerlinAnalytix GmbH, 12109 Berlin, Germany; 3 NMI Natural and Medical Sciences Institute, University of Tübingen, 72770 Reutlingen, Germany; 4 Beijing Advanced Innovation Center for Soft Matter Science and Engineering, College of Life Science and Technology, Beijing University of Chemical Technology, Beijing 100013, China; 5 Scientific Research Center for Biomedicine, Department for Cell and Tissue Engineering, Faculty of Medicine, University of Niš, 18000 Niš, Serbia; 6 Department of Biology and Human Genetics, Faculty of Medicine, University of Niš, 18000 Niš, Serbia; 7 Department of Traumatology and Orthopedics, Peoples’ Friendship University of Russia, 117198 Moscow, Russia; 8 University Medical Centre, Justus Liebig University of Giessen, 35390 Giessen, Germany

**Keywords:** absorbable membrane, polytetrafluoroethylene (PTFE), titanium, collagen sources, crosslinking, bioactive factor, immune response, macrophages, vascularization

## Abstract

Guided bone regeneration (GBR) has become a clinically standard modality for the treatment of localized jawbone defects. Barrier membranes play an important role in this process by preventing soft tissue invasion outgoing from the mucosa and creating an underlying space to support bone growth. Different membrane types provide different biological mechanisms due to their different origins, preparation methods and structures. Among them, collagen membranes have attracted great interest due to their excellent biological properties and desired bone regeneration results to non-absorbable membranes even without a second surgery for removal. This work provides a comparative summary of common barrier membranes used in GBR, focusing on recent advances in collagen membranes and their biological mechanisms. In conclusion, the review article highlights the biological and regenerative properties of currently available barrier membranes with a particular focus on bioresorbable collagen-based materials. In addition, the advantages and disadvantages of these biomaterials are highlighted, and possible improvements for future material developments are summarized.

## 1. Introduction

Guided bone regeneration (GBR), as one of the most common strategies for alveolar ridge preservation/augmentation, is regarded as a standard treatment modality. Barrier membranes play a key role in GBR by forming a barrier between soft tissue and the bone defect area, thus facilitating the proliferation of osteoprogenitor cells and supporting new bone tissue formation. In addition to the space maintenance function, a successful material design of the “ideal” GBR membrane should take into account the following properties: (1) biocompatibility: does not damage the surrounding tissue and the healing process; (2) cellular occlusion: prevents the invasion of non-osteogenic cells into bone defect from the mucosa; (3) easy handling: not too rigid without sacrificing space maintenance function; (4) bioactivation properties: promotes wound healing and tissue integration [1]. It is generally accepted that barrier membranes require 4–6 weeks of standing time for periodontal tissue regeneration and 16–24 weeks for bone enhancement [1,2,3].

A large number of commercially available barrier membranes have been reported [4,5,6]. This heterogenous group can be generally divided into two categories according to their degradability: non-resorbable and resorbable membranes.

The history of non-resorbable barrier membrane applied in GBR procedures dates back to the 1980s [7]. Initially, the first barrier membrane generation was conceived to function only as occlusive membranes. In 1982, Nyman et al. evaluated the performance of a millipore membrane in bone regeneration preclinically mediated by periodontal ligament cells [8]. Later in 1986, Gottlow et al. clinically implanted polytetrafluoroethylene (PTFE) in 10 patients, which is still the most commonly used non-absorbable membrane even until today [9]. In particular, the later development of expanded polytetrafluoroethylene (ePTFE) membranes (also in combination with titanium reinforcement) was accepted as the gold standard material for their mechanical stability and increased space maintenance capacity, biocompatibility, and efficacy to facilitate bone regeneration [10,11]. However, non-resorbable membranes have two serious limitations to clinical use. One limitation is their stiffness that can lead to soft tissue dehiscences, which can lead to membrane exposure and complications ultimately resulting in implant failure [12,13]. The second limitation is the need for a second surgery that must be performed to remove the non-resorbable membrane [14,15].

Based on these facts, a new membrane class including synthetic and naturally sourced absorbable membranes has been developed to overcome the shortcomings of non-absorbable membranes. Among these, collagen membranes are the most widely studied and clinically applied due to their superior biocompatibility and bioactivities such as chemotactic to the periodontal ligament (PDL) or gingival fibroblasts and strong adhesion of osteoblasts on the membrane surface [16,17]. However, the rapid degradation rates and the poor volume stability properties of most collagen barrier membranes are still important limiting factors [18]. Another important disadvantage of this material class is the rapid fragmentation and degradation after gingival dehiscence with membrane exposure and related decreased bone regeneration [2].Thus, various methods such as physical/chemical/enzymatic and crosslinking strategies such as ultraviolet (UV) radiation, genipin (Gp), and glutaraldehyde treatments have been analyzed, to extend both the degradation time and mechanical properties of collagen membranes for overcoming the current material deficiencies [19,20]. In addition, collagen membranes are often combined with different agents such as bone grafts or resorbable stabilizing structures such as magnesium meshes to increase their regenerative capacities and to prevent membrane collapse and volume stability [21,22]. A further focus for improvement of the performance of a resorbable barrier membrane is to have influence on their “bioactivity”, including factors such as transmembraneous vascularization or different approaches for “immunomodulating” properties such as influence on macrophage phenotypization [19,23]. In this context, an increasing number of preclinical studies are focusing on the loading of active compounds such as growth factors, cytokines, inorganic compounds, and anti-inflammatory agents among different others [3,24,25]. With the emphasis on membrane bioactivity, membrane-associated cellular and molecular events have gained much interest including cell recruitment, inflammation, and bone healing [26,27]. Although the mechanisms involved are still unclear, growing evidence offers the possibility of modulating the sequence and intensity of biological events through material properties. In this context, the aims of this review are: (a) to present on overview of the different types of barrier membranes used for GBR, (b) to highlight collagen sources and recent advances in collagen membrane modification, including crosslinking and loading with active factors, and (c) to summarize the biological mechanisms associated with collagen membranes.

## 2. Nonabsorbable Barrier Membranes

Despite the drawbacks of complications and secondary surgery faced by non-resorbable membranes, their space-making ability in combination with volume stability in the case of titanium-reinforced materials remains clinically irreplaceable, especially for large, non-contained bone defects, or vertical augmentations [11,28,29].

### 2.1. Polytetrafluoroethylene (PTFE)

**Figure 1:**
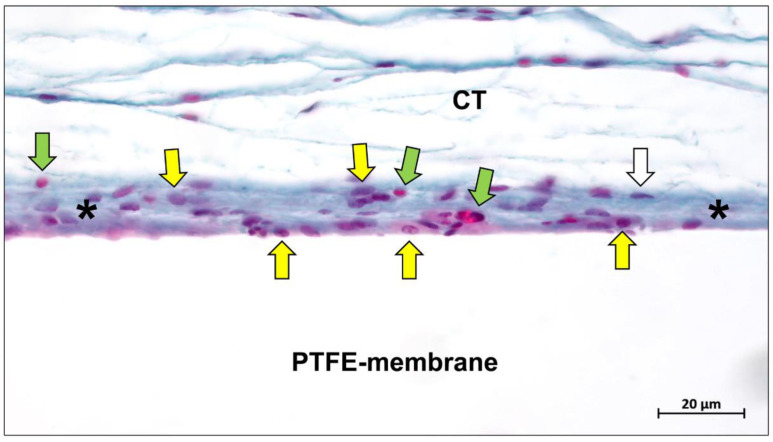
Representative histological image showing the tissue reaction to a subcutaneously implanted PTFE membrane at day 30 post implantationem. Asterisks = thin reactive tissue wall, yellow arrows = macrophages, green arrows = eosinophils, white arrow = fibroblast, CT = connective tissue (Alcian blue staining, 400× magnification, scalebar = 20 µm).

PTFE is the base material of the most representative and earliest clinically used non-absorbable membranes [7,30]. This material is based on an unbranched, linear, semi-crystalline polymer combining fluorine and carbon [31]. PTFE belongs to the class of polyhaloolefins, and to the so-called thermoplastics [32]. It is also considered to be very inert [33]. One reason is the particularly strong bond between the carbon and fluorine atoms. Thus, many substances are unable to break the bonds and react chemically with PTFE [31]. Furthermore, PTFE is kinetically inhibited by the compact shell of fluorine atoms that protects the inner carbon strand [32]. It is therefore extremely resistant to all bases, alcohols, ketones, etc. [34]. Moreover, PTFE has a very low coefficient of friction [35]. No materials exist that will stick to PTFE because the surface tension is extremely low [36]. This inert material is difficult to wet and almost impossible to bond [37]. However, Korzinskas and colleagues showed that PTFE-based barrier membranes induce a slight (inflammatory) tissue reaction comparable to collagen-based materials (Figure 1).

#### 2.1.1. e-PTFE and d-PTFE

Different representative PTFE membranes have been developed according to different clinical requirements:

1. expanded polytetrafluoroethylene (e-PTFE);
2. high-density polytetrafluoroethylene (d-PTFE).

Biomaterials based on e-PTFE are fabricated via extrusion of PTFE molecular fibers, resulting in two different fiber orientations, i.e., mono- and multidirectional materials [31,38]. The production of monodirectional e-PTFE results in a material whose molecular fibers are oriented in one direction, while multidirectional e-PTFE, on the other hand, is oriented in many directions [39]. This creates a complex fiber structure that gives the material exceptional strength and creep resistance in both longitudinal and transverse directions [40]. Furthermore, its production in combination with a lubricant results in a microporous material structure characterized by connection nodes that are interconnected by longitudinal fibrils of less than 0.5 μm in diameter. The degree of porosity of an e-PTFE material can be controlled by the distance between the nodes [31].

The porous structure of e-PTFE facilitates nutrient transport and has proven its clinical stability and excellent biocompatibility [15,28]. Although there is still controversy regarding the relationship between membrane type and exposure rate [14,41], a clinical systematic evaluation showed a significantly higher incidence of membrane exposure for non-absorbable membranes (20%) than for absorbable membranes (5%) [42]. A meta-analysis of membrane exposure noted that areas without membrane exposure achieved 74% higher horizontal bone gain than areas with membrane exposure in edentulous ridges [12]. Conventional e-PTFE membranes demonstrated a weak barrier effect against bacterial infection after membrane exposure, which increases the difficulty of postoperative care and the risk of bone regeneration failure [41]. In contrast, dense polytetrafluoroethylene (d-PTFE) is a less porous form of polytetrafluoroethylene, which has also been on the market for many years, especially in combination with titanium grids for an enhancement of the volume stability (see next paragraph). Although both PTFE membranes showed similar clinical outcomes in the treatment of peri-implant vertical bone defects [28], the dense structure of d-PTFE is considered to be effective in preventing bacterial invasion while retaining the potential for oxygen diffusion and small molecule transport [43]. In addition, postoperative removal of the d-PTFE membrane is easier than that of the e-PTFE membrane, which is essential for subsequent recovery and overall healing [28]. However, the accumulation of thicker biofilms on d-PTFE membranes observed in some studies suggests that bacterial infection appears to be related not only to porosity. In vitro evaluation of a novel bilayer e-PTFE membrane with optimized layer thickness as well as the extent and direction of swelling by Trobos et al. showed better resistance to bacterial permeability and biofilm formation than d-PTFE [39]. This conclusion is also supported by clinical randomized studies where more biomass accumulation and thicker biofilms were observed on d-PTFE membranes [38].

#### 2.1.2. Titanium-Reinforced PTFE-Membranes

Although e-PTFE and d-PTFE have demonstrated adequate barrier function and space maintenance, the osteogenic spaces they create are prone to deformation under pressure in the face of large vertical clinical bone defects, which is detrimental to bone regeneration [44]. Titanium-reinforced membranes have been created to overcome this deficiency. A titanium skeleton with high strength and stiffness is inserted into the PTFE membrane to give it excellent plasticity and volume stability [45]. Ti struts not only provide excellent mechanical support but also allow for easy clinical placement under the flap [46]. Currently, titanium-reinforced membranes are an established core material for providing volume-stable osteogenic space to promote bone tissue regeneration in clinical procedures. In a recent meta-analysis, titanium-reinforced d-PTFE supporting the highest vertical bone regeneration with a low complication rate was considered to be the best choice for GBR/GTR [13].

### 2.2. Titanium Meshes and Cages

Titanium is a popular metal material in dentistry and other medical fields. In particular, its clinical suitability for GBR procedures is unmatched by other GBR membranes. Traditionally, titanium meshes have been used clinically, primarily to stabilize the bone grafts and maintain the desired bone morphology and volume rather than providing a barrier functionality, as this material class does not have a space maintenance function due to the large pore size [47]. Due to their exceptional volume stability, titanium meshes are indispensable in the management of vertical or large horizontal bone defects. Titanium meshes not only demonstrate high strength and stiffness but also exhibit good plasticity, allowing them to be perfectly adapted to various bone defects through bending and shaping [48,49]. Although the sharp edges created by cutting, trimming, or bending of titanium mesh can raise concerns about membrane exposure, titanium mesh has a significantly lower postoperative exposure rate than most barrier membranes and does not usually need to be removed immediately because infection does not usually occur after exposure [50,51]. Based on these advantages, a systematic clinical evaluation of titanium meshes for alveolar bone reconstruction noted a mean success rate of 89.9%, a mean survival rate of 100%, and a failure rate of 0% [52]. To further improve the clinical suitability of titanium meshes, digitally tailored titanium mesh technology has been used in the clinic. The titanium meshes manufactured by 3D printing and other technologies are often imaginatively referred to as titanium cages, which allow for a perfect fit to the bone defect and avoid the undesirable consequences of incorrect placement [53,54]. A clinical study involving 40 patients (65 implant sites) demonstrated that digital titanium mesh significantly reduced postoperative vertical and horizontal bone resorption and performed well in maintaining hard tissue stability. In this investigation, the exposure rate of digital titanium meshes was only 10%, which is lower than the exposure rate reported in most previous studies [55].

The combination of titanium mesh + PTFE membrane or titanium mesh + collagen membrane is usually used in clinical practice, where the PTFE or collagen membrane provides space maintenance, and the titanium mesh provides volume stability [56,57,58]. Notably, a recent case study involving 106 patients with perioral repair showed that collagen membrane coverage prevents peripheral tissue adhesion, fibrosis, and associated problems, which are common inflammatory responses to titanium mesh [59]. This proven combination strategy means that space maintenance is not clinically necessary for titanium mesh application. More systematic histological evidence is therefore needed to elucidate the effect of titanium mesh structure (thickness and porosity) on bone healing mechanisms to guide the manufacture of clinically meaningful titanium meshes.

## 3. Absorbable Barrier Membrane

The advantage of absorbable barrier membranes over non-absorbable membranes is the absence of later surgical removal. At the same time, absorbable membranes face the challenge of matching the material absorption with the period of tissue regeneration. The rapid degradation of the membrane and its potential degradation of byproducts often leads to clinical failure of guided bone regeneration [15]. Typically, the inefficient volume stability caused by the low mechanical strength of absorbable membranes compared to non-absorbable membranes is the most significant clinical limitation [60].

Depending on their origin, absorbable membranes are usually divided into natural polymers, represented by collagen and synthetic polymers represented by aliphatic polyesters (e.g., poly (lactic acid) (PLA), poly (polyglycolic acid) (PGA), poly (ε-caprolactone) (PCL)) [35,46]. Synthetic polymers are highly customizable, allowing for precise control of barrier membrane shape, thickness, porosity, mechanical strength, and degradation properties by adjusting chemical structure and preparation conditions [61,62]. Based on these advantages, the development of synthetic polymer membranes has been the focus of the next generation of barrier membranes. Although most synthetic polymers are considered non-cytotoxic and degradable, synthetic polymer membranes still have the pitfalls of strong inflammatory reactions caused by oligomers and acidic byproducts released during degradation, as well as the degradation period of some polymers (e.g., PCL) that is too long (2–3 years) for GBR procedures [46,62].

The most striking feature of natural polymeric membranes is their inherent bioactivity, which results in high biocompatibility and a more beneficial tissue regeneration microenvironment [35]. At the same time, natural polymers pose new problems involving partially strong immunogenic reactions, complex purification processes, and the risk of disease transmission [63]. Collagen and chitosan are two of the most representative natural polymeric membranes that have attracted the most research, with collagen in particular dominating the clinical landscape.

As the predominant component of the extracellular matrix, collagen exhibits excellent biocompatibility due to its structural support and regenerative properties [16]. Among more than twenty collagen types that have been classified, type I and type III collagen from porcine and bovine tissues are the predominant sources of biomaterials [3]. Collagen has many properties that make it suitable for GBR procedures. In addition to the advantages of a single-step procedure, collagen membranes clinically accelerate early wound stabilization and initial closure of the defect [64,65]. Collagen membranes also show low exposure rates, especially when compared to non-absorbable membranes, and their rapid absorption after exposure also effectively eliminates the open microenvironment for bacterial infection [66,67,68]. Furthermore, concerning its biological properties, collagen is the only animal-derived barrier membrane material whose low immunogenicity as well as adhesion and chemotaxis to fibroblasts and osteoblasts can mediate excellent tissue integration and angiogenesis [60,63,69]. Collagen membranes have also been shown to adsorb bone and cell-released active factors (e.g., TGF-b) as a molecular mechanism that contributes to bone regeneration [70,71,72]. Collagen membranes have therefore attracted a lot of interest as biomaterials with unique bioactive functions.

Many different types of collagen membranes have been developed and are used in clinical practice, such as Bio-Gide^®^ (Geistlich Pharma AG, Wolhusen, Switzerland), Jason^®^ (botiss biomaterials GmbH, Zossen, Germany), Ossix^®^ (Datum Dental, Lod, Israel), and Periogen^®^ (Collagen Corporation, Palo Alto, CA, USA). Due to the different collagen sources and extraction processes, these collagen membranes show different chemical and physical structures.

**Figure 2:**
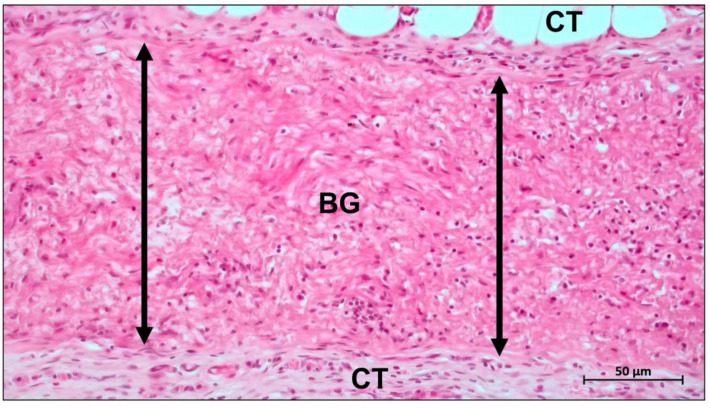
Representative histological image showing the tissue reaction and integration behavior of the Bio-Gide membrane (GB, double arrows) within the subcutaneous connective tissue (CT) at day 60 post implantationem (HE staining, 200× magnification, scalebar = 50 µm).

The most important commercial collagen membrane Bio-Gide^®^ is composed of porcine-derived type I and type III collagen. It contains a bilayer structure consisting of a dense layer and a porous layer, which enables the regulation of barrier function [73]. This bilayer structure supports the migration and differentiation of osteoblasts while preventing the invasion of fibroblasts, making it a classic solution for the structural design of barrier membranes [35]. The dense layer of Bio-Gide^®^ remains intact up to 60 days after implantation, while the porous layer is completely degraded (Figure 2) [74].

A similar structural design can also be achieved by crosslinking. For example, He et al. showed that the large pore size (240–310 μm) oxidized sodium alginate (OSA) crosslinked collagen membranes had the greatest promotion of osteogenic differentiation, while the small pore size (30–60 μm) membranes had the greatest improvement in barrier function [75]. The bilayer membrane obtained by combining the two pore sizes exhibited good osteogenesis promotion and barrier function to fibroblasts [75]. Moreover, different materials can also be taken to design multilayer membranes. For example, a fish collagen and polyvinyl alcohol (Col/PVA) bilayer membrane was developed by Zhou et al. [76]. The PVA layer provided sufficient mechanical support, and the collagen layer not only showed good cytocompatibility but also promoted the expression of osteogenic genes (RUNX2, ALP, OCN, and COL1) and proteins (ALP) in BMSCs.

**Figure 3:**
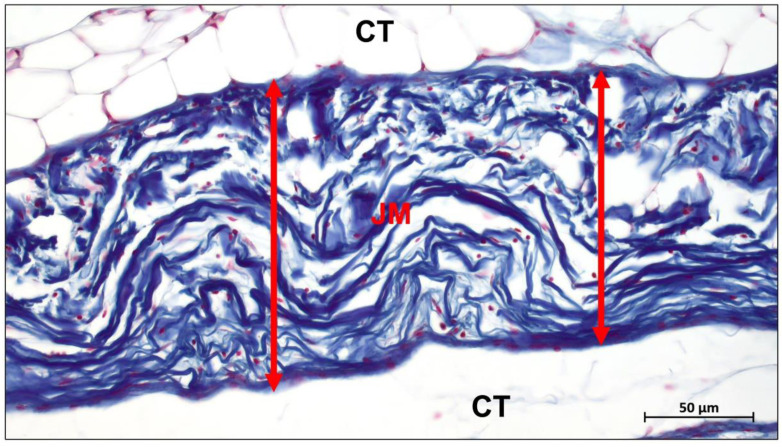
Representative histological image showing the tissue reaction and integration pattern of the pericardium-based barrier membrane Jason (JM, double arrows) within the subcutaneous connective tissue (CT) at day 120 post implantationem (Masson Trichrome staining, 200× magnification, scalebar = 50 µm).

Jason membrane is the second most clinically used collagen membrane. This barrier membrane is derived from porcine pericardium and is known for its excellent long barrier function (8–12 weeks) (Figure 3) [77]. It exhibits excellent multidirectional tear resistance due to the preservation of the natural collagen structure of the pericardial tissue during the manufacturing process [78]. Jason membrane is very thin, only 0.05–0.35 mm thick, and therefore does not swell after rehydration [79]. The internal structure of the membrane exhibits a natural crosslinked honeycomb-like collagen arrangement, thus demonstrating slow degradation [74].

**Figure 4:**
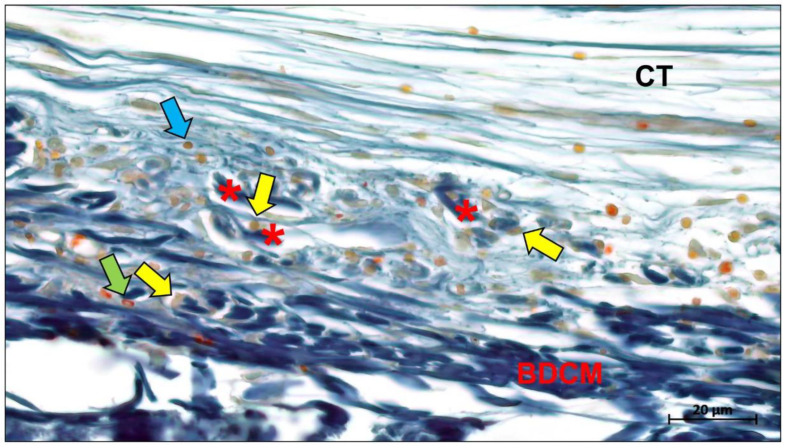
Representative histological image showing the inflammatory tissue reaction to a bovine dermis-derived collagen membrane (BDCM) within the subcutaneous connective tissue (CT) undergoing cellular degradation at day 60 post implantationem. Red asterisks = membrane fragments, yellow arrows = macrophages, green arrow = eosinophil, blue arrow = lymphocyte (Azan staining, 400× magnification, scalebar = 20 µm).

The main factors currently limiting collagen membranes are their low stiffness and rapid degradation in vivo. After implantation, collagen membranes can be absorbed by enzymatic degradation mediated by collagenases, bacterial proteases, and macrophage-derived enzymes (Figure 4) [80,81].

**Figure 5:**
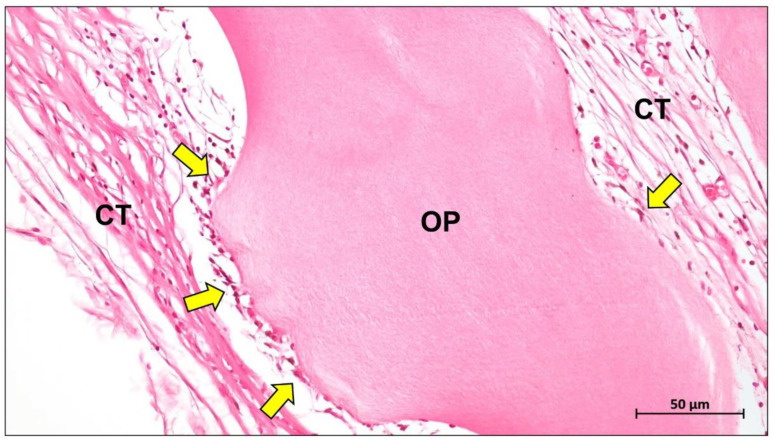
Representative histological image showing the tissue reaction to the ribose crosslinked barrier membrane Ossix Plus (OP) within the subcutaneous connective tissue (CT) at day 60 post implantationem. Yellow arrows = macrophages (HE staining, 200× magnification, scalebar = 50 µm).

Modification of collagen membranes is therefore a necessary path. Crosslinking offers promising solutions for improving the mechanical strength of collagen membranes. The commercially available Ossix ^®^Plus, for example, is a very densely structured ribose crosslinked membrane that is absorbed after eight months in vivo (Figure 5) [74].

In addition, collagen membranes have shown the potential to further improve regenerative properties as effective carriers of active factors. Modification of collagen membranes will be described in detail in the next section.

### 3.1. Modification of Collagen Membranes

#### 3.1.1. Tissue Sources of Collagen Membranes

Collagen from different sources differs greatly in structure and composition, which also greatly affects the cellular response and degradation pattern of collagen membranes in vivo [16,82]. Altogether, conclusions can be made about the origin of the tissue and the resultant properties of the barrier membrane. The main collagen currently available is mainly from pericardium, and skin of mammals with a high homology to human collagen [82,83]. Of these, skin and tendon are favored due to their high collagen content [84].

**Figure 6:**
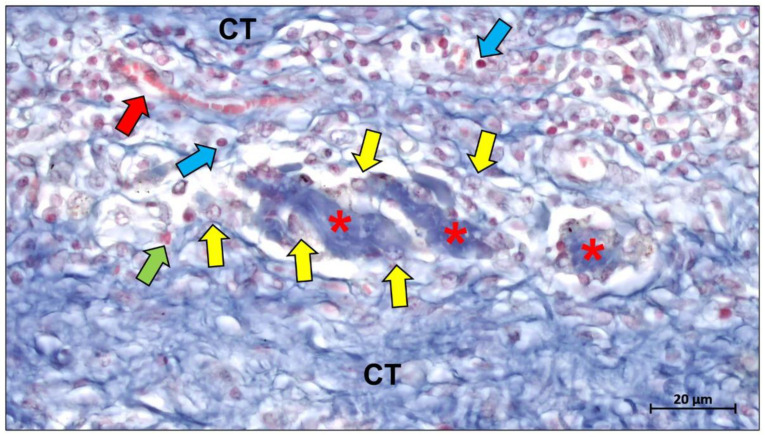
Representative histological image showing the fast biodegradation of a native dermis-derived collagen material (red asterisks = material remnants) within the subcutaneous connective tissue (CT) at day 15 post implantationem. Yellow arrows = macrophages, blue arrows = lymphocytes, green arrow = eosinophilic granulocyte, red arrow = blood vessel (Masson Goldner staining, 400× magnification, scalebar = 20 µm).

Mammalian dermis contains up to 60–70% collagen, and its fibers are anisotropically distributed along the longer line [85]. The collagen fibers in the dermis are arranged in a loose network and contain a mixture of macromolecules such as hyaluronic acid, dermatan sulfate and chondroitin sulfate, which in combination with a large amount of water, fill the spaces between the collagen fibers [82]. In addition, the skin tissue contains a large number of blood vessels, lymphatic vessels, hair follicles and sweat glands, so that the purification step is more demanding [86]. Although native porcine collagen membranes showed temporal differences in their biodegradation pattern, it seems that this membrane type degrades faster compared to membranes based on materials won from other tissue or animal sources (Figure 6) [87].

**Figure 7:**
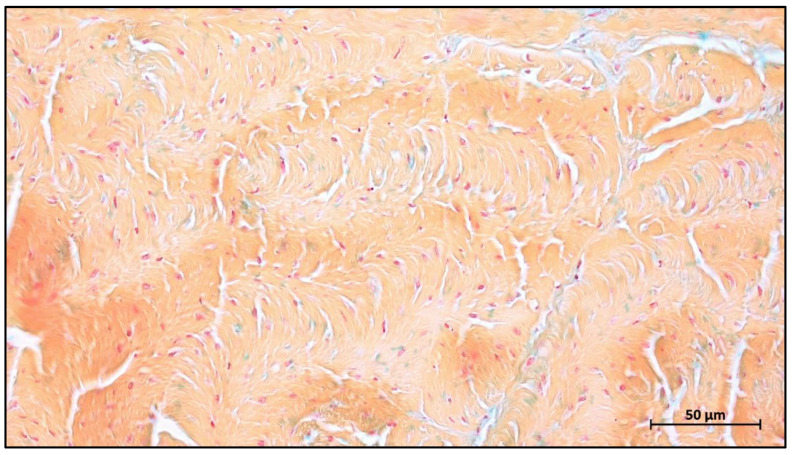
Representative histological image showing native porcine Archilles tendon tissue (prior to decellularization) with a high portion of collagen (yellow staining) and contained tendocytes (red staining) (Movat Pentachrome staining, 200× magnification, scalebar = 50 µm).

The collagen of tendon accounts for up to 85% of its dry weight and is composed almost entirely of type I collagen [88,89]. Therefore, this area has the lowest level of collagen contaminants and is the best site for type I collagen extraction. In addition, the structure of the tendon allows the collagen fibers to curl and align in the main load bearing direction (Figure 7) [83]. Even after the extraction procedure, collagen fibers from tendon retain lateral stacking arrangement [90]. The above theory supports the hypothesis that devices derived from the Achilles tendon have a higher chemical–physical stability. However, collagen membranes derived from tendon are rare on the market due to the high cost. Biocollagen^®^ (Bioteck by Bioteck SpA, Torino, Italy), a collagen membrane derived from the equine Achilles tendon, has also not shown a satisfactory degradation pattern, with a standing time of only 4–6 weeks [91], which seems to contradict the initial hypothesis. However, this rapid degradation could also be attributed to the species; thus, more evidence is still needed to clarify the degradation pattern of the device derived from the Achilles tendon.

Collagen from the pericardium is also attractive in terms of mechanical properties because it retains the biomechanical properties of the pericardial tissue [92]. It often exhibits excellent multidirectional tearing resistance due to the inclusion of fine, wavy and multidirectional-oriented collagen fibers within [93]. Moreover, the natural crosslinking degree seems to increase the standing time, as it has been revealed that such materials are still providing barrier functionality up to 12 weeks post implantationem [74].

**Figure 8:**
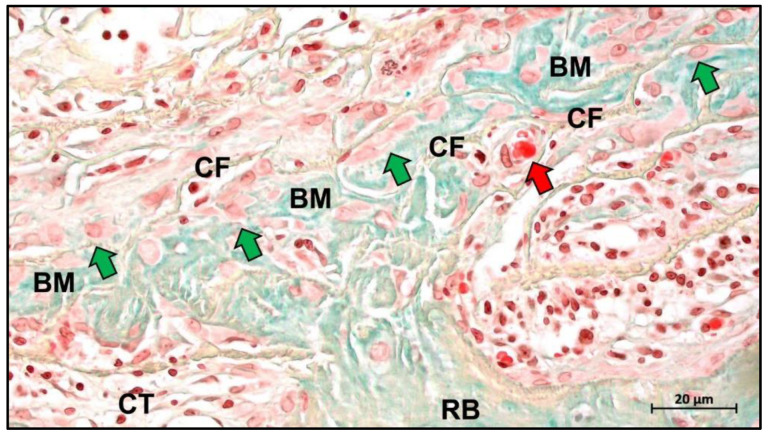
Representative histological image showing the bone ingrowth into a pericardium-based collagen membrane at 2 weeks post implantationem. BM = newly formed bone matrix within the membrane, green arrows = osteoblasts, red arrow = blood vessel, CF = collagen fibers of the membrane, RB = residual bone, CT = connective tissue (Movat Pentachrome staining, 400× magnification, scalebar = 20 µm).

Moreover, it was shown in a preclinical study by Alkildani et al. that a pericardium-based barrier membrane was completely ossified over a period of 16 weeks and thus contributes to bone tissue regeneration in combination with a bone substitute material (manuscript in preparation) (Figure 8).

Among mammals, bovine and porcine are the most important sources of collagen extraction because they are the most consumed meat *per capita* [94]. However, it is still partly assumed that they might face exaggerated immune reactions and transfer infectious diseases beside different religious restrictions [95]. Collagen from horses is considered to be generally free of infectious disease risks and immune reactions [96]. Equine collagen also has the highest homology to human collagen similar to that of bovine collagen, making it an effective alternative to bovine collagen [94]. Equine tendon collagen has been reported to have higher levels of lysine and hydrogen lysine compared to other mammalian tendon, making its biologics theoretically more resistant to degradation and tear resistance [84,97]. This hypothesis is highly controversial, however, as Toledano et al. showed that of the three collagen membranes tested, collagen membranes from porcine dermis had the greatest resistance to degradation compared to equine pericardium and equine lyophilized collagen felt [98]. Similar results were confirmed by Vallecillo-Rivas et al. that equine collagen membranes showed the weakest degradation resistance among the five membranes tested [91]. Equine meat and its biological products are not accepted by Jews and Muslims.

In this context, non-mammalian marine organisms become a very attractive alternative source [99]. Jellyfish can contain up to 60% collagen, which is homologous to mammalian collagen types I, II and V; hence, the term type 0 collagen [100]. Jellyfish collagen has been shown to induce higher fibroblast and osteoblast viability than bovine collagen [101,102]. Although barrier membranes from jellyfish are not currently available on the market, a study by Flaig et al. showed that jellyfish collagen scaffold (Jellagen^®^-3D scaffolds, Jellagen^®^ Ltd., Cardiff, UK) induced an overall weaker immune response than porcine pericardial collagen scaffolds. It induced long-term M2 cell responses and optimal vascularization patterns within the implantation bed [103].

#### 3.1.2. Collagen Crosslinking Strategies

Poor mechanical properties and high degradation rates of native collagen-based barrier membranes are still dominant limitations in clinical applications. To enhance durability and mechanical strength, crosslinking as an efficient method has attracted extensive studies. In principle, crosslinking inhibits sliding between collagen molecules under pressure by introducing intramolecular and intermolecular covalent or non-covalent bonds, which increases the stiffness, tensile strength, compressive modulus, and reduced extensibility of collagen fibers [19]. At the same time, intermolecular crosslinking also improves the resistance of collagen against enzymatic degradation by masking the cleavage site of collagen [104]. Crosslinking of collagen can be induced by a variety of techniques (shown in Table 1), which are briefly reviewed in the following.

##### Physical Strategies

Physical crosslinking methods of collagen mainly comprise dehydrothermal (DHT) and ultraviolet (UV) irradiation. Both processes do not require the incorporation of any chemical agents avoiding the threat of biological toxicity.

UV induces the formation of highly reactive free radicals, which mediates the formation of intra- and extra-fibrillar carbonyl-based covalent bonds at aromatic amino acid residues [20]. This method is highly germicidal, as the UV light destroys the genetic material of microorganisms [105]. However, UV-induced collagen denaturation, which counteracts the stabilizing effect, occurs continuously during crosslinking [106]. The presence of water in UV crosslinking is necessary to initiate free radical production, and the rate and amount of free radical production is the main limiting factor for crosslinking degree [107]. It has been stated that high crosslinking densities cannot be achieved by UV induction alone [105]. Combination with light-activated reagents, such as riboflavin as the source of reactive oxygen species, appears to be more effective. In 2019, Zhang et al. fabricated a UVA/riboflavin crosslinking amniotic membrane, which exhibited bamboo-like structural changes accompanied by increased brittleness and stiffness, further prolonging the degradation rate in vivo [108]. UV crosslinking strategies based on photoactive atelocollagen precursors have also been developed recently. Liang et al. designed atelocollagen functionalized with 4-vinyl benzyl chloride (4VBC) and methacrylic anhydride (MA) to fabricate a UV-cured GBR membrane. MA as the highly reactive monomer has been widely proofed to generate crosslinked networks rapidly through photo-induced free radicals [109]. Furthermore, 4VBC-functionalized collagen was demonstrated to exhibit significantly increased compression properties compared to methacrylated collagen [110]. The introduction of 4VBC was also found to inhibit the activity of matrix metalloproteinases (MMPs) [111]. The resultant membrane showed excellent compressibility, swelling ratios, and increased proteolytic stability with respect to Bio-Gide^®^ as one of the most common commercial collagen membranes. Altogether, the UV crosslinking membrane is mainly limited by the difficulty of achieving satisfactory mechanical properties compared to chemical crosslinking. It is therefore often used as an auxiliary crosslinking method, in combination with other crosslinking methods. In addition, UV crosslinking is more suitable for fabricating thin or transparent membrane considering the penetration of UV irradiation.

Dehydrothermal (DHT) is another common physical method for collagen crosslinking that involves exposure to high temperatures in a vacuum condition to cause the formation of intermolecular amide and ester bonds through dehydration [112]. As early as 1996, it was shown that DHT-induced crosslinking exhibits a higher contraction temperature of collagen fibrils while reducing their solubility in solutions containing collagenase in comparison to UV crosslinking [113]. The temperature and the period of this treatment are the main regulators of crosslinking degree [20]. It is well known that the crosslink density increases with temperature and time within a certain range [114]. However, excessive temperatures and processing periods can lead to disruption of the triple helix structure of collagen, which usually deteriorates the mechanical properties [115]. It is now generally accepted that the optimum temperature for DHT is around 140–150°. Chen et al. demonstrated that the mechanical properties of collagen membranes were severely impaired when the DHT treatment temperature exceeded 145° or the treatment time exceeded 5 days [116]. However, considering the foreign body reaction after material implantation, the optimal treatment time may be further reduced. The study by Nakada et al. showed that excessive heat treatment results in little to no cellular infiltration of the collagen material and a large number of foreign body giant cells around the material, ultimately resulting in no tissue regeneration [117]. In addition, their study in 2017 showed that thermal treatment at 140 °C for 6 h supports new bone formation and gingival fiber regeneration, which is considered to be a favorable outcome for GBR [118]. This result is also supported by the study of An et al. where DHT membranes showed better enzymatic resistance and tensile strength than Bio-Gide^®^ besides exhibiting well tissue integration in vivo [119]. Thus, DHT shows the potential to produce barrier membranes, especially to handle thick materials that are difficult to treat with UV.

##### Chemical Strategies

**Figure 9:**
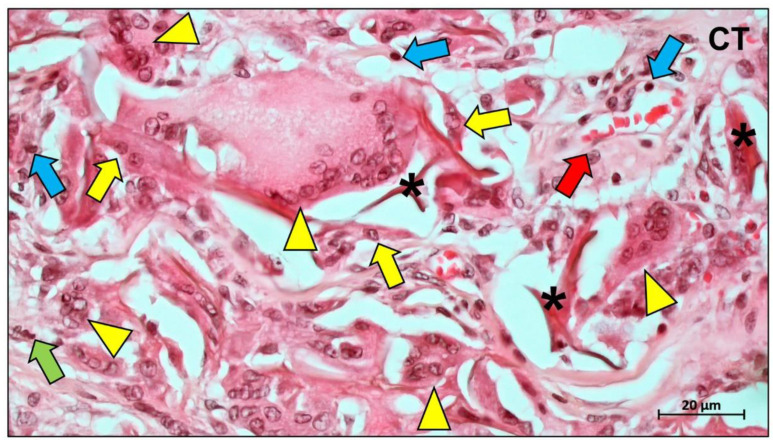
Representative histological image showing the strong inflammatory tissue reaction to a GA crosslinked collagen material (asterisks = material remnants) within the subcutaneous connective tissue (CT) at 15 days post implantationem. Yellow arrows = macrophages, yellow arrowheads = multinucleated giant cells, blue arrows = lymphocytes, green arrow = eosinophilic granulocyte, red arrow = blood vessel, (HE staining, 400× magnification, scalebar = 20 µm).

Glutaraldehyde (GA) is the most widely used traditional crosslinking agent for collagen, due to its low cost, high reactivity, and high solubility in aqueous solutions. However, it has been rarely used for tissue engineering studies in recent years due to its local cytotoxicity and induction of calcification as well as inflammatory responses (Figure 9) (Shi 2020).

In contrast, 1-Ethyl-3-(3-dimethylaminopropyl)carbodiimide/N-hydroxysuccinimide (EDC-NHS) is a “zero-length” crosslinker that chemically activates the carboxylic acid groups of aspartic acid and glutamic acid on collagen to conjugate with hydroxylysine and amines of lysine residues through direct covalent bonds without any linkers or spacers [20,106]. All residues of this method are water-soluble and therefore can be easily washed out from the material by distilled water after crosslinking [120]. DHT/EDC crosslinked collagen membranes exhibit good enzyme resistance, mechanical properties, and excellent peripheral tissue integration compared to commercially available noncrosslinked collagen membranes (Bio-Gide^®^) in a rat subcutaneous model [119,121]. The bone regeneration ability of EDC/NHS crosslinked membranes has also been demonstrated both in the Beagle mandible model and the rabbit calvaria defects model [121]. However, recent studies on the interaction properties of EDC/NHS-modified collagen with cells show that both the affinity and pattern of cellular interactions are modulated by carbodiimide treatment. The study by Bax et al. seems to indicate that the depletion of carboxyl groups of glutamic acid on collagen by EDC/NHS crosslinking hinders its binding to integrins and thus reduces integrin-mediated cell adhesion. Furthermore, with increasing EDC concentration, the cell adhesion pattern to collagen shifts from divalent metal cation-dependent binding to metal ion non-dependent binding [122].

Natural crosslinkers have significant advantages over traditional chemical crosslinkers in terms of biocompatibility. Genipin is the best-known natural crosslinking agent mainly extracted from the fruits of *Gardenia jasminoides* L. or *Gardenia jasminoides* [123]. It is only 0.01% as cytotoxic as glutaraldehyde and exhibits significant anti-inflammatory properties [124]. Nair et al. showed that collagen membranes crosslinked by Genipin provided higher viability of human dermal fibroblasts than EDC/NHS crosslinked membranes [125]. However, Genipin is currently limited by its high cost in mass production and is mainly used in laboratory studies. He et al. prepared a bilayer collagen membrane with controlled pore size and significantly improved mechanical strength by crosslinking with sodium oxide alginate (OSA) [75]. This study showed that OSA crosslinking significantly improved the compressive strength and swelling properties of collagen membranes and prolonged the degradation period [75]. With increasing OSA content, the residual mass ratio increased from 14.9% to 59.0% after 21 days in vitro enzymatic degradation [75]. Natural polyphenols are also widely used as natural crosslinkers for collagen with excellent anti-inflammatory, anti-bacterial and anti-cancer properties [126]. Proanthocyanidins (PAs) or tannins are the most common flavonoid natural polyphenolic compounds and are widely considered to be effective in stabilizing collagen [127,128,129]. With a highly hydroxylated structure, PAs can form strong hydrogen bonds with soluble collagen making it a good candidate for crosslinking agents. Incorporation of grape seed proanthocyanidins into dental adhesives can promote the enzymatic resistance of collagen at the dentin/adhesive interface and significantly prolong the degradation period of collagen fibers [128]. PAs also showed significant inhibition and anti-adhesion against principal cariogenic bacteria (Streptococcus mutans and Lactobacillus acidophilus) [130] and microorganisms causing oral infections (*Enterococcus faecalis*, *Porphyromonas gingivalis*, and *Clostridium difficile*) [131,132]. In addition, oligomeric proanthocyanidins (OPCs) released from dentin can induce differentiation of dental pulp cells (DPC) to a phenotype favoring biomineralization [133]. The collagen membrane crosslinked with procyanidins developed by Yang et al. could well block the migration of WS-1 and MG-63 cells. In vitro experiments showed that the proliferation, differentiation, and mineralization of MG-63 cells were promoted on the resultant membrane [134]. This is consistent with the study by Li et al. that OPCs crosslinked collagen membranes support the proliferation of L929 and MG-63 cells, in addition to exhibiting up to 50 days of standing time and supporting new bone growth in vivo [135]. Epigallocatechin-3-gallate (EGCG), which has a high structural similarity to PAs, is another commonly used natural crosslinking agent. EGCG crosslinked collagen membranes significantly downregulated the level of inflammatory factors secreted by MG63 cells in in vitro experiments. However, higher concentrations of EGCG showed a slight inhibition of cell viability [136]. The incorporation of polyethylene glycol (PEG) into the collagen membrane of EGCG could offset the dose limitation to some extent [137]. EGCG crosslinked small intestinal submucosa (E-SIS) also showed enhanced adhesion of fibroblasts and pro-osteoblasts and promoted osteogenic differentiation of MC3T3-E1 cells cultured on E-SIS. The E-SIS membrane also accelerated bone regeneration in a rat cranial defect model (Gou 2019). Furthermore, studies by Chu et al. and Rung et al. showed that EGCG crosslinked collagen membranes facilitate the recruitment of macrophages [136,138]. Notably, EGCG modification has a strong ability in promoting vascularization involving the secretion of M2-related cytokines [136].

##### Enzymatic Strategies

The crosslinking and stability of collagen in vivo largely depend on enzymatic reactions. The most representative one is the transglutaminase that catalyzes the formation of ε-(γ-glutaminyl)-lysine isopeptide bonds to assemble various proteins related to mineralized tissue formation (e.g., collagen, fibronectin, osteopontin, and bone sialoprotein) into polymeric forms involved in matrix stabilization, chondrocyte and osteoblast differentiation, and matrix mineralization [139]. Natural type I collagen treated with tissue transglutaminase (TG2) and microbial transglutaminase (mTG) has been shown to enhance the adhesion, spreading, and proliferation of human osteoblasts (HOB) and human foreskin dermal fibroblasts (HFDF). It also exhibited increased endogenous protease resistance and differentiation rate of HOB cells [140]. Fortunati et al. showed similar results and proposed a mechanism by which TG2-modified collagen induced enhanced osteoblast adhesion mediated by promoting integrin expression in human osteoblasts [141]. The microbial transglutaminase (mTGase) crosslinked HA/Coll scaffold also showed significantly improved thermal stability and compression modulus. mTGase modification also increased the adhesion, proliferation, and differentiation of MG63 osteoblast-like cells and human umbilical vein endothelial cells (HUVEC) on the scaffolds [142]. Enhanced differentiation of human mesenchymal stem cells to osteoblasts was also observed on microbial transglutaminase crosslinked tilapia scale collagen scaffolds and collagen type XI scaffolds [143,144]. Yang et al. compared gelatin sponges prepared by different crosslinking methods, where mTG crosslinking showed the best comprehensive performance in terms of mechanical strength and biocompatibility [145]. Although enzymatic crosslinking provides theoretically optimal biocompatibility and biomimetic properties, its improvement of mechanical properties is much lower than chemical crosslinking. Coupled with the low economic benefits associated with high costs, mass production applications of enzymatic crosslinking are unpractical in the short term.

**Table 1:** Recent developments of crosslinked collagen membranes for guide bone regeneration (GBR).

Author	Membranes Tested	Crosslinking Agents	Study Design	Mechanical Properties	Enzyme Resistance	Cell Cultivation	Osteogenesis and Organizational Integration Properties
Wang et al., 2022 [146]	Collagen/polycaprolactone methacryloyl/magnesium (Col/PCLMA/Mg) composite membrane	UV irradiation	In vivo and vitro	Increased elastic modulus, reduced swelling rate	Increased		Enhanced osteogenic capability
Wu et al., 2022 [147]	Chemical crosslinking collagen membrane combined with zinc-doped nanohydroxyapatite (nZnHA)	Glutaraldehyde-alendronate	In vivo and vitro	Increased tensile modulus and extreme tensile strength	Increased	Noncytotoxic	Improved tissue integration and vascularization
He et al., 2022 [75]	Chemical Crosslinking collagen bilayer membrane	Oxidized sodium alginate (OSA)	In vivo and vitro	Improved structural stability, compressive strength, swelling behavior	Increased		Osteogenic differentiation was most promoted on the membrane with a large pore size (240–310 μm)
Yang et al., 2021 [134]	Chemical Crosslinking collagen membrane	Oligomeric proanthocyanidins (OPCs)	In vitro			Promoted osteoblast proliferation and differentiation	
Liang et al., 2021 [148]	Sequentially functionalized atelocollagen membrane	UV irradiation	In vivo and vitro	Improved compressive strength, swelling behavior	Increased	Increased metabolic activity	Enough safety, occlusivity, and soft tissue barrier functionality
Hong et al., 2021 [149]	Chemical and physical crosslinking collagen membrane	Carbodiimide, biphasic calcium phosphate (BCP)-supplemented UV irradiation	In vivo		Increased		Chemical crosslinking increased inflammatory response, both chemical and physical crosslinking distinctively enhanced new bone formation in the early phase of healing.
Rung et al., 2021 [138]	Chemical crosslinking collagen membrane	EDC/NHS and EGCG	In vivo and vitro	Moderately enhanced stiffness, slightly weakened elasticity.		Promoted cell viability, adhesion, and vessel formation	Upregulated angiogenesis-related factor VEGF, downregulated microphages markers F4/80.
Zhao et al., 2020 [150]	Chemical crosslinking collagen bilayer membrane	Dialdehyde carboxymethyl cellulose	In vitro	Improved tensile strength, reduced swelling behavior	Increased	Good blood compatibility and cytocompatibility	Enhanced alkaline phosphatase (ALP) activity, promoted the differentiation of MC3T3-E1 cells.
Zhang et al., 2020 [108]	Amniotic membrane	UVA/riboflavin	In vivo and vitro	Increased brittleness and hardness	Increased		Enhanced resistance to tissue dissolution
Ahn et al., 2020 [121]	Chemical crosslinking collagen membrane	EDC	In vivo and vitro	Improved tensile strength	Increased	Noncytotoxic	Similar bone regeneration compared with noncrosslinking membrane.
Li et al., 2019 [135]	Chemical crosslinking collagen membrane	Oligomeric proanthocyanidins (OPCs)	In vivo and vitro	Improved thermal stability and tensile modulus	Increased	Noncytotoxic but even promote L929 cells growth	Good tissue integration
Guo et al., 2019 [151]	Chemical crosslinking small intestinal submucosa membrane	Epigallocatechin-3-gallate (EGCG)	In vivo and vitro	Improved ultimate stress (US), elastic modulus (EM)		Enhanced the adhesion of fibroblasts and pre-osteoblasts, and promoted the osteogenic differentiation of MC3T3-E1 cells	Accelerated bone regeneration
Russo et al., 2019 [152]	Porcine pericardium membrane	Polyphenol-rich pomace extract (PRPE)	In vitro	Improved stiffness and Young’s modulus	Increased		
Muñoz-González et al., 2018 [153]	Chemical crosslinking collagen membrane	Trifunctional oligourethane	In vitro	Increased relaxation time	Increased	Imparted capacity to modulate macrophages	
An et al., 2018 [119]	Physical and chemical crosslinking collagen membrane	Dehydrothermally (DHT) and DHT/EDC	In vivo and vitro	Increased tensile strength	Increased		Promoted Angiogenesis and tissue integration
Wei et al., 2018 [154]	Chemical crosslinking collagen membrane loading β-TCP	Oligomeric proanthocyanidins (OPCs)	In vitro	Increased compression modulus	Increased	Promoted the proliferation of MG-63 cells	

#### 3.1.3. Incorporation of Bioactive Molecules

The binding of multiple bioactive molecules to membranes has received the most attention because of their multifunctional role in osteogenesis, particularly cell recruitment, proliferation, and differentiation [3]. Commonly used bioactive molecules are summarized in Table 2 and presented in this section.

##### Cytokines and Growth Factors

The binding of active molecules to the membrane is based on the hypothesis that exposure of the treated area to multiple different growth factors can trigger the development of a favorable microenvironment and promote bone regeneration [25,155]. Platelet-derived growth factor (PDGF) is a potent mitogenic and chemo-inductive agent, and in particular, PDGF-BB is more effective than other isoforms such as PDGF-AA and PDGF-AB in promoting mitosis in periodontal cells [156]. The development of recombinant PDGF-BB (rhPDGF-BB) has given momentum to its use in bone regeneration. rhPDGF-BB also showed potent mitogenic, angiogenic and chemotactic effects on bone and periodontal cells [157]. The binding of rhPDGF-BB to collagen membranes has been shown to release 60% of the factor within the first three days, followed by a sustained release in vitro for approximately 3 weeks [158]. Recently, Joshi et al. demonstrated in a clinical study that PDGF-BB can be loaded on collagen membranes and released slowly for up to 1 month at sites of intraosseous defects [159]. Bone morphogenetic proteins (BMP) are also bioactive molecules in the field of bone regeneration and have been considered in several reviews to be the most promising growth factor for bone regeneration [155,160]. Among the various isoforms, BMP-2 and BMP-7 are thought to play an important role in osteogenic differentiation [161]; especially, BMP-2 has been shown to induce both cartilage and sclerogenesis [162,163]. Even low doses of rhBMP-2 (0.2 mg/mL) loaded in the Hydroxyapatite/β-tricalcium phosphate/Collagen (HAp/TCP/Col) complex exhibit strong osteogenic potential in the Beagle dog model [164]. However, some recent studies seem to suggest that BMP-9 possesses a stronger osteoinductive potential than BMP-2 [165,166]. Saulacic et al. showed that in a rabbit cranial defect model, BMP-9 loaded on collagen membranes induced better horizontal bone defect closure than loading on deproteinized bovine bone mineral, and both combinations positively induced bone regeneration [77,167]. The significant bone-promoting potential of the combination of BMP-9 and collagen membranes has also been demonstrated in an anti-resorptive therapy (AMART) mouse model [25]. In other studies, stromal cell-derived factor-1α (SDF-1α) has been widely accepted to promote the recruitment, proliferation, and differentiation of bone marrow-derived stromal stem cells (BMSCs) as a classical chemokine, mediating significant bone regeneration and angiogenesis [129,168]. The combination of SDF-1 and basic fibroblast growth factor (bFGF) plays an active role in BMSCs-mediated periodontal membrane regeneration, as they induce BMSCs to differentiate into cells with periodontal membrane fibroblast characteristics [169,170]. In the subject of active compound delivery, the way it is attached to the GBR membrane is fundamentally important for in situ tissue regeneration. Yu et al. showed that collagen membranes chemically conjugated to SDF-1α significantly promoted the formation of new bone and microvessels compared with SDF-1α physisorption and showed a similar effect on new bone formation to the BMSC seeding method [170].

Although all these studies demonstrate the feasibility of delivering active molecules through membranes, the instability (e.g., conformational changes and degradation) and early burst release of protein factors greatly hinder their clinical application. The supraphysiological dose of active molecules to compensate for their instability also raises toxicity and cost concerns [171]. Delivery of DNA or RNA encoding the relevant regenerative factor rather than its protein form is a promising solution. Non-viral vector delivery of pDNA encoding PDGF-B on a collagen scaffold was demonstrated in a rat cranial defect model with a significant increase in new bone volume/total volume (BV/TV) % (14-fold and 44-fold higher) compared to empty defects or empty scaffolds, respectively [172]. Synergistic delivery of pDNA encoding FGF-2 and BMP-2 also shows significant improvement in bone regeneration in diaphyseal long bone radial defects [173]. Delivery of chemically modified ribonucleic acid (cmRNA) may be more efficient and safer because the absence of nuclear trafficking can effectively improve transfection efficiency [174]. Elangovan et al. demonstrated that the polyethylenimine (PEI)-cmRNA (encoding BMP-2) complex promoted significantly enhanced bone regeneration compared to PEI-DNA (encoding BMP-2) [172]. The team also reported that collagen sponges containing cmRNA (BMP-9) had stronger bone regeneration efficacy than those containing cmRNA (BMP-2), with a two-fold higher junctional density of regenerated bone [173]. The functionality of pDNA (BMP-9) and cmRNA (BMP-9) integration into collagen membranes was also confirmed in vitro and in vivo, with enhanced osteogenic differentiation and bone volume fraction [23].

##### Metal Ions

Many studies have been conducted to demonstrate that the doping of trace elements, such as zinc (Zn), magnesium (Mg), cobalt (Co), and strontium (Sr), can further enhance the biological activity of collagen. In addition, the accompanying release of trace elements can regulate the local biological environment. The interest in the osteogenic properties of Sr stems from the therapeutic effects of strontium ranelate in osteoporosis. Sr supports osteoblast differentiation and activates the expression of one of the osteoblast markers, osteopontin, a marker of late osteoblasts [175]. Sr also has a concomitant inhibitory effect on bone resorption by osteoclasts [176,177]. In this dual mode of action, Sr creates a favorable environment for bone tissue remodeling and healing. Furthermore, in vitro studies with human mesenchymal stem cells (MSCs) have shown that Sr-doped matrices are not cytotoxic, regardless of the amount of Sr incorporated [178]. Zn is also known to be a potent bone immunomodulator, affecting macrophage polarization and osteoblast differentiation [179]. Wu et al. showed that 1% and 2% nZnHA-doped collagen membranes exhibited superior biocompatibility and stronger promotion of multinucleated giant cells (MNGC) formation in vitro and in vivo [147]. On the other hand, zinc seems to modulate the biological activity of matrix metalloproteinases (MMP) to regulate collagen degradation [180]. There is also evidence that both transforming growth factor-β (TGF-β) and osteoprotegerin (OPG) are upregulated when osteoblasts are exposed to zinc ions [181]. Metal–organic framework (MOF) crystal-modified electrospun asymmetric bilayer polycaprolactone/collagen (PCL/Col) membranes further achieved PH-responsive release of zinc ions, showing enhanced osteoinductivity and angiogenesis both in vitro and in vivo [24]. The important role of magnesium in maintaining bone strength and bone formation makes it promising for bone regeneration therapy [182,183]. Magnesium has been shown in vitro to have a promotive effect on a variety of bone cells [184]. For example, it enhances the proliferation and migration of human osteosarcoma MG-63 cells and alkaline phosphatase (ALP) activity [185], promotes integrin α2 and α3 mediated proliferation, and enhances ALP expression and activity in bone marrow-derived stromal cells (hBMSC) [186]. The above evidence suggests a beneficial role of trace elements in bone tissue regeneration, which can be considered as effective bioactive modulators without cytotoxicity. However, the effects of metal ions are usually concentration-dependent and should still be considered with caution until sufficient clinical confirmation is obtained.

##### Antimicrobials and Antibiotics

Antibacterial agents and antibiotics, such as tetracyclines [27], metronidazole [187], and silver ions, are added mainly to prevent bacterial infections especially when membrane exposure occurs. In vitro studies have shown that AgNP-coated collagen membranes show excellent antibacterial efficacy against *Staphylococcus aureus* (*S. aureus*) and *Pseudomonas aeruginosa* (*P. aeruginosa*) without obvious cytotoxicity. The AgNP-coated membrane also has effective anti-inflammatory effects by inhibiting the expression and release of anti-inflammatory cytokines such as IL-6 and TNF-α. In addition, the resultant membrane was able to induce osteogenic differentiation of mesenchymal stem cells, demonstrating its osteogenic potential [188]. Amoxicillin-loaded poly (D, L-lactic acid) membranes implanted in vivo show an early reduction in inflammation and accelerate periodontal repair [189]. Polysaccharide membranes containing gentamicin also support osteoblast growth [190]. In particular, Ghavimi et al. recently developed an asymmetric GBR membrane benefiting from curcumin and aspirin reported a striking ability to promote bone regeneration [191]. The asymmetric membrane achieved complete bone regeneration after 28 days in the animal test, while the area of commercial membrane remained empty. The above results seem to indicate that the addition of antimicrobial agents and antibiotics is also beneficial for bone and tissue regeneration rather than just anti-infection. However, its effects on cells and tissues are highly dose-dependent. Xie et al. showed that PMMA membranes loaded with relatively low concentrations of vancomycin (1–4 g/cement dose) can slightly promote osteoblast viability and angiogenesis [192]. In contrast, relatively high vancomycin concentrations (6–10 g/cement dose) showed decreased osteoblast viability and reduced angiogenesis. In addition, although many antimicrobial strategies have been developed in in vitro and in vivo experiments, there are widespread concerns about the risk of multi-resistant bacterial strains associated with the overuse of antibiotics, especially broad-spectrum antibiotics, making it difficult to assess the clinical safety of antimicrobial strategies. In this dilemma, the development of novel natural antimicrobial agents seems to be necessary. Among them, antimicrobial peptides (AMP) offer new possibilities for this idea because of their difficulty in causing bacterial resistance and the versatility that can be obtained through flexible amino acid sequence design. Zhou et al. designed antimicrobial peptides containing osteogenic fragments attached to AgNP through hydrogen bonding [193]. Peptide rods covered with AgNPs@AMP functional coating promote osteogenic gene expression (ALP, COL 1, β-Actin, OCN and Runx-2) and osseointegration in vivo.

**Table 2:** Recent advances of barrier membranes in combination within bioactive molecules.

Characteristics	Modification	Author	Experimental Groups	Main Funding
Loading of growth factors or cytokines	PDGF	Nevins 2003 [157]	rhPDGF-BB incorporated in bone allograft	Purified rhPDGF-BB mixed with bone allograft results in robust periodontal regeneration in both Class II furcations and interproximal intrabony defects.
		Yamano 2011 [158]	rhPDGF-BB incorporated in CM	PDGF significantly increased gene expression of osteoblast differentiation markers and ALP and cell proliferation activities with little cytotoxicity in MC3T3-E1 cells.
		Joshi 2019 [159]	Platelet-Rich-Fibrin (PRF) membrane or CM incorporated with rhPDGF-BB	Both PRF membrane and CM incorporated with rhPDGF-BB showed comparable gingival crevicular fluid (GCF) levels of PDGF-BB initially, with PRF showing more sustained levels throughout the study period.
		Elangovan 2014 [172]	pDNA encoding PDGF-B on a collagen scaffold	A significant increase in new bone volume/total volume (BV/TV) % (14-fold and 44-fold higher) compared to empty defects or empty scaffolds, respectively.
	BMP	Chao 2021 [164]	rhBMP-2 loaded in the HAp/TCP/Col complex	HAp/TCP/Col with 0.2 mg/mL rhBMP-2 manifested strong osteogenic potential with more and faster new bone formation and better implant stability in Beagle dog model.
		Saulacic 2017 [167], Fujioka-Kobayashi 2017 [77]	BMP-9 loaded on CM. BMP-9 loading on deproteinized bovine bone mineral	BMP-9 loaded on collagen membranes induced better horizontal bone defect closure than loading on deproteinized bovine bone mineral, and both combinations positively induced bone regeneration.
		Khorsand 2017 [173]	PEI-(pBMP-2+pFGF-2) embedded in collagen scaffolds. PEI-pBMP-2 embedded in collagen scaffolds	Synergistic delivery of pDNA encoding FGF-2 and BMP-2 also shows significant improvement in bone regeneration in diaphyseal long bone radial defects.
		Elangovan 2015 [172]	PEI-pPDGF-B complex-loaded collagen scaffold	The PEI-cmRNA (encoding BMP-2) complex promoted significantly enhanced bone regeneration compared to PEI-DNA (encoding BMP-2)
		Khorsand 2017 [194]	cmRNA (BMP-9) loaded collagen sponges. cmRNA (BMP-2) loaded collagen sponges.	cmRNA (BMP-9) had stronger bone regeneration efficacy than cmRNA (BMP-2), with a two-fold higher junctional density of regenerated bone.
		Khorsand 2019 [23]	pDNA (BMP-9) loaded CM. cmRNA (BMP-9) loaded CM.	Calvarial bone defects treated with CM-cmRNA(BMP-9) trended toward being higher than defects treated with CM-pDNA(BMP-9) and CM alone.
	SDF-1α	Yu 2020 [195]	Physical adsorption group with Bio-Oss+SDF-1αphysically adsorbed on the CM. Chemical crosslinking group with Bio-Oss+SDF-1α chemically crosslinked to the CM	Collagen membranes chemically conjugated to SDF-1α significantly promoted the formation of new bone and microvessels compared with SDF-1α physisorption and showed a similar effect of new bone formation to the BMSC seeding method.
Loading of metal ions	Sr	Ehret 2017 [178]	Strontium-doped hydroxyapatite polysaccharide materials	Sr-doped matrices are not cytotoxic in vitro, regardless of the amount of Sr added. In vivo, subcutaneous implantation of these Sr-doped matrices induced a transformation of bone tissue and blood vessels.
	Zn	Wu 2022 [147]	nZnHA-doped collagen membranes	1% and 2% nZnHA-doped collagen membranes exhibited superior biocompatibility and stronger promotion of multinucleated giant cells (MNGC) formation in vitro and in vivo.
		Chou 2016 [181]	zinc hydroxyapatite loaded gelatin membrane	Both transforming growth factor-β (TGF-β) and osteoprotegerin (OPG) are upregulated when osteoblasts are exposed to zinc ions.
		Xue 2021 [24]	PCL/Col/ZIF-8 Composite Membrane	PCL/Col/ZIF-8 composite membrane achieved PH-responsive release of zinc ions, showing enhanced osteoinductivity and angiogenesis both in vitro and in vivo.
Loading of antimicrobials and antibiotics	Silver nanoparticle	Chen 2018 [196]	Silver nanoparticle-coated collagen membrane	The AgNP-coated membrane also has effective anti-inflammatory effects by inhibiting the expression and release of anti-inflammatory cytokines such as IL-6 and TNF-α. In addition, the resultant membrane was able to induce osteogenic differentiation of mesenchymal stem cells.
	Amoxicillin	Ho 2021 [189]	Amoxicillin loaded poly (D, L-lactic acid) membrane	Early reduction in inflammation and accelerate periodontal repair in vivo.
	Gentamicin	Cibor 2017 [190]	Gentamicin loaded Polysaccharide membrane	Resultant membrane support osteoblast growth and show favorable pharmacokinetics, bactericidal activity, cytocompatibility and good mechanical properties.
	Curcumin Aspirin	Ghavimi 2020 [191]	Nanofibrous asymmetric collagen/curcumin membrane containing aspirin loaded PLGA nanoparticles	The asymmetric membrane achieved complete bone regeneration after 28 days in animal test.
	Vancomycin	Xie 2022 [192]	Vancomycin loaded PMMA membranes	PMMA membranes loaded with relatively low concentrations of vancomycin (1–4 g/cement dose) can slightly promote osteoblast viability and angiogenesis.
	Antimicrobial peptide	Zhou 2022 [193]	AgNPs@AMP functionally coated peptide rods	AgNPs@AMP functional coating promote osteogenic gene expression (ALP, COL 1, β-Actin, OCN and Runx-2) and osseointegration in vivo.

## 4. Biological Mechanisms of Collagen Membrane

Barrier membranes have been shown to provide more than just barrier functionality in GBR procedures [197]. For example, not only collagen membranes but even non-resorbable PTFE membranes have been shown to stimulate the expression of a variety of osteogenic-related genes (e.g., alkaline phosphatase (ALP), bone bridging proteins, and osteosalivary proteins), bone remodeling genes, and inflammatory cytokines (interleukin (IL)-6 and IL-1) [26,198]. This triggered the initial hypothesis that a barrier membrane applied during GBR applications form a specific microenvironment under the membrane to support the migration and later differentiation of osteoblasts [199]. Studies on collagen membranes have revealed a molecular mechanism that provides partial evidence for a submembraneous cell recruitment mechanism, as the presence of collagen membranes causes the early upregulation of two cell recruitment factors (CXC chemokine receptor type 4 (CXCR4) and monocyte chemotactic protein-1 (MCP-1)) [70]. CXCR4 plays a key role in the recruitment of osteogenic progenitor cells and mesenchymal stem cells, which subsequently differentiate into osteoblasts and participate in bone formation [200,201], while MCP-1 is a main chemokine in the recruitment of osteoclast progenitor cells, a key cell type in bone remodeling [201]. The above evidence suggests that the membrane promotes a microenvironment at the defect site that favors the rapid recruitment of different cells, including osteoblasts and osteoclasts, which further promotes a molecular cascade that facilitates remodeling for bone formation.

**Figure 10:**
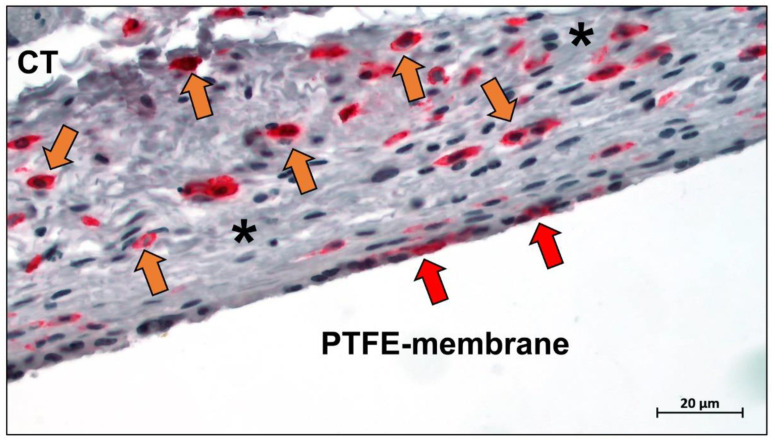
Representative histological image showing the pro-inflammatory tissue reaction (orange arrows = pro-inflammatory macrophages within the surrounding connective tissue, red arrows = pro-inflammatory macrophages at the material surface) to a PTFE-membrane at 30 days post implantationem. Asterisks = reactive cell wall, CT = connective tissue (CD11c immunostaining, 400× magnification, scalebar = 20 µm).

Although the current wealth of histological evidence relating to bone healing and regeneration beneath barrier membranes is insufficient to explain the clear role of barrier membranes in defect healing involving inflammation, cell recruitment, and bone remodeling, it has been shown that the immune response elicited by different materials is specific, depending on the physicochemical properties of the material [202]. This “foreign body response to biomaterial” begins with the rapid accumulation of proteins on the surface of the material after implantation and occurs in almost all types of biomaterials [203]. It has been shown that even PTFE membranes, which are completely biologically inert, can induce an immune response in vivo (Figure 10) [43].

This raises the key question of whether the foreign body response triggered by the membrane is necessary and beneficial for tissue regeneration at the defect. This is based on the fact that the immune response appears to mediate the degradation of the material and transmembrane vascularization.

**Figure 11:**
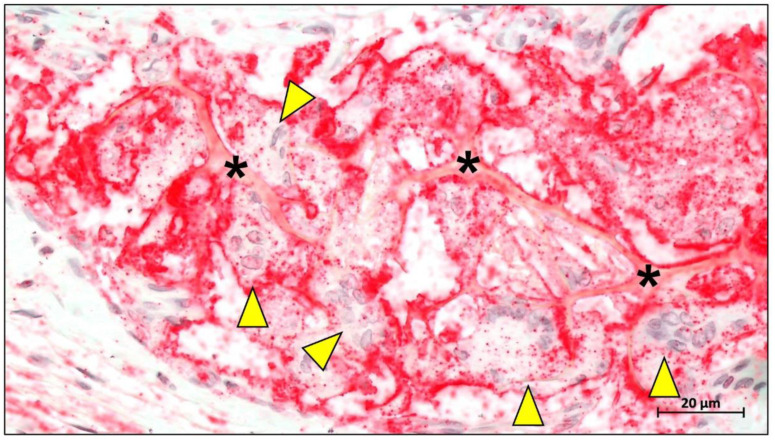
Representative histological image showing strong pro-inflammatory tissue reaction (red staining) mainly involving multinucleated giant cells (yellow arrowheads) to a GA crosslinked collagen membrane (asterisks = material remnants) within the subcutaneous connective tissue at 2 weeks post implantationem. (CD11c immunostaining, 400× magnification, scalebar = 20 µm).

The inflammatory tissue response is a cascade effect. Inflammatory cells such as monocytes, macrophages, and neutrophils direct the pattern of the immune response cascade through their interaction with proteins and the release of cytokines [204,205]. Within this cascade, macrophages play a key regulatory role involving the transformation of cell types. Depending on their molecular expression, macrophages can be divided into two phenotypes: the pro-inflammatory M1 phenotype and the anti-inflammatory M2 phenotype [206]. The M1 phenotype occurs mostly in the early stages of tissue healing and appears to mediate the in vivo degradation of the material. Subsequently, during the acute inflammatory remission phase, the M1 phenotype is polarized to the M2 phenotype, which primarily expresses reparative factors [207,208]. It is therefore generally accepted that excellent tissue regeneration must be accompanied by an overall M2 tissue response, but it is also important to be wary of failed tissue remodeling due to fibrous encapsulation from chronic inflammation [208,209]. Multinucleated giant cells (MNGCs) are a type of immune cell with greater phagocytic capacity resulting from the fusion of macrophages. Notably, MNGCs have been shown to be of the foreign body giant cell phenotype rather than the traditionally thought osteoclasts [210,211]. Similar to macrophages, MNGCs also express pro- and anti-inflammatory factors on the implantation bed and therefore also appear to exhibit a pro-inflammatory M1-BMGCs phenotype and anti-inflammatory M2-BMGCs phenotype (Figure 11) [208,210]. In addition, MNGCs have been shown to mediate the phagocytic degradation of different materials [212,213,214,215,216].

Although the relationship between the different material properties and the activation and expression patterns of MNGCs is unclear, it has been established that the immune response is crucial in the bone healing process. Modification of material properties can optimize the immune response of biomaterials to better support bone healing.

**Figure 12:**
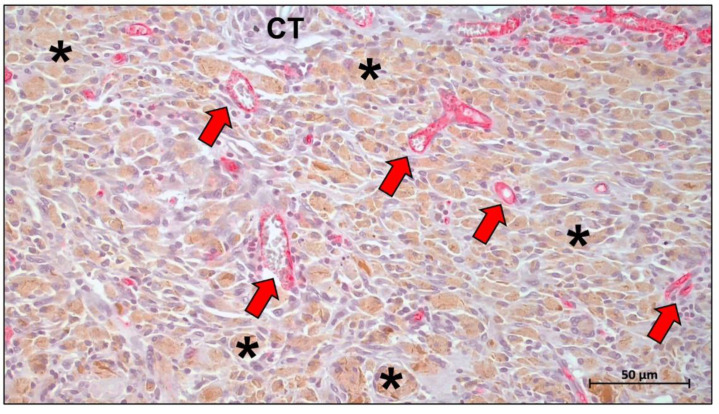
Representative histological image showing vessel ingrowth (red arrows and red staining) into a dermis-derived collagen membrane (brown staining and asterisks) at 30 days post implantationem. CT = connective tissue (CD31 immunostaining, 200× magnification, scalebar = 50 µm).

Angiogenesis is an important part of the microenvironment that facilitates bone healing. The concept of “transmembrane vascularization” has been proposed for collagen barrier membranes [217]. Previous studies have shown that successful tissue integration of different porcine liver-derived collagen membranes does not require transmembrane vascularization to be mediated but is associated with the induction of granulation tissue [87,218]. Nevertheless, transmembrane vascularization remains attractive for achieving better regenerative outcomes (Figure 12).

Barbeck et al. evaluated two porcine dermal-derived collagen membranes (Mucoderm and Collprotect) and noted that although neither membrane showed successful transmembrane vascularization, the Mucoderm membrane containing the vascular backbone allowed for microvascular penetration associated with the inward growth of connective tissue [87]. This provides evidence for the hypothesis that transmembrane vascularization must be based on the inward growth of connective tissue. However, this hypothesis seems to contradict the underlying function of the barrier membrane, as successful transmembrane vascularization in this situation would imply membrane fragmentation and disintegration and thus failure to perform the barrier function. Recent studies based on bovine collagen membranes seem to offer a solution. Histopathological results of bovine collagen membranes suggest that bovine membranes undergo macrophage and multinucleated giant cell-mediated fragmentation mostly around 60 days after implantation [219]. However, the membrane did not completely lose its shielding function, as the fragments overlapped in a tile-like arrangement under the connective tissue. Reactive tissue could penetrate the membrane through the interstices between the fragments and substantial vascularization occurred. This particular pattern of integration and degradation is not observed in porcine collagen membranes and has been defined by researchers as “secondary porosity” [219,220].

In conclusion, understanding the biological mechanisms of membranes and the related integration pattern in vivo is essential for material preparation. Once the relationship between different material properties and mechanisms related to bone remodeling is clearly understood, material properties can be used to regulate key biological events to support better tissue regeneration.

## 5. Conclusions

The use of barrier membranes to block the inward growth of soft tissue is a standard strategy that has been used successfully in clinical practice. Resorbable and non-resorbable membranes differ in terms of clinical procedures, complication rates, and long-term outcomes. Despite the need for secondary surgery, non-resorbable membranes are noted to be irreplaceable in clinical situations dealing with large or vertical bone defects because of their excellent mechanical and barrier properties. The most common complications of non-resorbable membranes are early membrane exposure and subsequent bacterial infections. Titanium mesh demonstrates superior clinical performance to PTFE membranes in this regard. Not only is the clinical exposure rate significantly lower than that of PTFE, but it also supports successful tissue regeneration after exposure has occurred. In recent years, the development of emerging technologies, such as digitally planed and patient-specific titanium meshes, has made non-resorbable membranes highly tailorable in clinical settings. Of the many resorbable membranes, collagen membranes are dominant in clinical practice due to their well-established scientific background and extensive clinical validation. The unique biological properties of collagen membranes and the absence of secondary surgery are considered potential candidates for the ideal barrier membrane.

The source of collagen has been shown to be an important factor influencing cellular responses and membrane degradation patterns. Mammals remain the primary source of extracted collagen. Collagen membranes from the pericardium show greater resistance to tearing and longer degradation cycles. However, marine sources of collagen (e.g., jellyfish collagen) have gained much attention in recent years due to the absence of infectious disease and religious factors involved, showing favorable bone regeneration immune response and vascularization patterns.

Modification of collagen membranes is necessary and effective for achieving better clinical tissue regeneration, generally by crosslinking and carrying bioactive molecules. The initial hypothesis of crosslinking is to improve the mechanical strength and degradation cycle of collagen membranes and thus influence the clinical outcome of collagen membranes. Various chemical/physical/enzymatic crosslinking methods have been developed to successfully prepare crosslinked collagen membranes. However, the high degree of crosslinking of collagen fibers is associated with a higher exposure rate and sometimes affects the foreign body reaction during resorption. The loading of bioactive molecules is mainly based on the hypothesis of constructing an optimal microenvironment to increase bone remodeling. Most of the loaded active molecules undergo two phases: explosive release and slow release. Their effect on inducing tissue regeneration is usually concentration-dependent, and therefore, more clinical evidence is needed to verify the optimal loading concentration.

The biological mechanisms of collagen membranes are not yet fully defined. However, the importance of membrane bioactivity has been repeatedly highlighted. Increasing histological evidence showed that the foreign body response of collagen membranes in vivo is closely associated with macrophages and multinucleated giant cells. A comprehensive understanding of the molecular mechanisms and cellular responses associated with tissue healing of different membranes has important implications for regulating bone regeneration through material properties. On the other hand, it is unclear whether different membranes have similar cellular responses and molecular mechanisms in different hosts. Collectively, this review summarizes the basics of barrier membranes for GBR, focusing on advances in collagen membrane modification and their biological mechanisms. The summarization and synthesis of this information are essential to guide the development of the next generation of barrier membranes.

## Data Availability

Not applicable.
